# Retroperitoneal Teratoma in an Adult: A Potential Pitfall in the Differential Diagnosis of Adrenal Myelolipoma

**DOI:** 10.1155/2016/5141769

**Published:** 2016-10-20

**Authors:** Madoka Kataoka, Hiroshi Fukushima, Yasukazu Nakanishi, Minato Yokoyama, Nobuaki Funata, Toru Motoi, Ken-ichi Tobisu, Fumitaka Koga

**Affiliations:** ^1^Department of Urology, Tokyo Metropolitan Cancer and Infectious Diseases Center Komagome Hospital, 3-18-22 Honkomagome, Bunkyo-ku, Tokyo 113-8677, Japan; ^2^Department of Pathology, Tokyo Metropolitan Cancer and Infectious Diseases Center Komagome Hospital, 3-18-22 Honkomagome, Bunkyo-ku, Tokyo 113-8677, Japan

## Abstract

We report a 32-year-old female case of a right adrenal gland mass detected on CT scan at medical checkup. CT and MRI showed a mass of 5.1 cm made of fat and calcification in the right adrenal gland, leading to the clinical diagnosis of adrenal myelolipoma. Because of its relatively large size and the patient's desire, the patient underwent gasless single-port retroperitoneoscopic adrenalectomy using the RoboSurgeon system. Histopathological examination revealed that the cystic tumor is composed of keratinized epidermis, mature fat, nerve, cartilage, bone, and sebaceous glands compressing the normal adrenal gland, leading to the diagnosis of retroperitoneal mature cystic teratoma. The patient remains free of recurrence 29 months after surgery. Retroperitoneal teratoma is relatively rare but clinically important because of high possibility of malignancy. In a case of an adrenal mass difficult to clinically distinguish retroperitoneal teratoma from adrenal myelolipoma, surgical resection via a minimally invasive approach would be the best therapeutic option.

## 1. Introduction

An adrenal mass containing fat, cysts, and calcification on imaging studies is occasionally encountered in clinical practice and is usually diagnosed with myelolipoma. Here, we report a case of such a right adrenal mass, in which an unexpected pathological diagnosis was made following surgical resection via a minimally invasive approach. The current case highlights limitations of image-based diagnosis of adrenal masses and the diagnostic and therapeutic role of minimally invasive surgery for them.

## 2. Case Presentation

**Figure 1:**
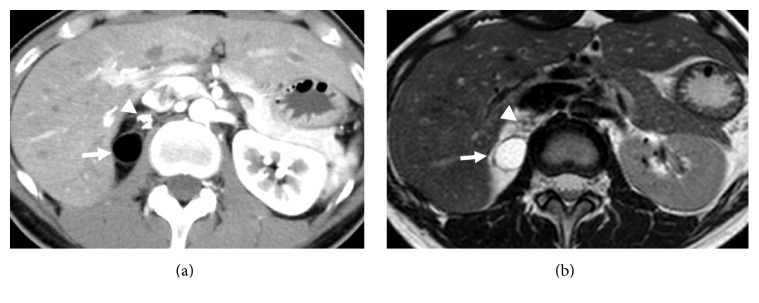
CT and MRI images of the tumor. Fat (white arrow) and calcification (white arrowhead) are shown in axial CT (a) and T2-weighted MRI (b) images.

A 32-year-old female patient presented with an incidental right adrenal mass pointed out on CT scan at medical checkup. The patient had no medical history. Results of complete blood count and routine biochemistry tests were normal. The endocrine function and tumor markers were also within normal ranges. According to imaging studies including CT and MRI (Figure 1), a right adrenal mass of 5.1 × 3.6 × 3.4 cm was composed of fatty density area and calcification. Thus, clinical diagnosis of adrenal myelolipoma was made based on radiological findings.

Because of its relatively large size and the patient's desire, she underwent gasless single-port retroperitoneoscopic adrenalectomy using the RoboSurgeon system [1]. The tumor was excised via a single port of 3 cm at the right flank through a retroperitoneal approach. Intraoperative bleeding was minimal, and the total duration of the operation was 144 minutes. No adverse events occurred postoperatively and she was discharged on day 5.

**Figure 2:**
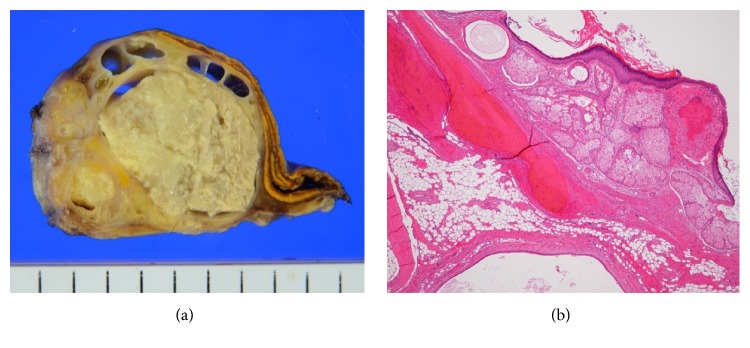
(a) Macroscopic findings of the resected tumor. The tumor contained cystic and solid components with adipose tissue. The right adrenal gland is compressed by the tumor. (b) Microscopically, the wall of the cystic component was lined by keratinized squamous epithelium and a mixture of mature components including sebaceous glands, cartilage, and bone in the solid component.

Macroscopically, the resected tumor contained multicystic and solid components including fatty tissue. The right adrenal gland was compressed but was not destructed by the tumor (Figure 2(a)). Microscopically, the wall of the cystic component was lined by keratinized squamous epithelium, and the solid component of the tumor was composed of a mixture of various mature components such as fat, nerve, cartilage, bone, and sebaceous glands (Figure 2(b)). Surgical margin was negative. Neither immature nor malignant components were identified. The tumor was pathologically considered as a mature cystic teratoma arising in the retroperitoneum adjacent to the right adrenal gland. She remains free of recurrence for 29 months after surgery.

## 3. Discussion

Among a fat-containing adrenal mass, the most common diagnostic entity is adrenal myelolipoma. In the current case, clinical diagnosis of adrenal myelolipoma was made based on radiological findings. Unexpectedly, histopathological diagnosis was retroperitoneal teratoma.

Teratoma is composed of plural germ layers: ectoderm, mesoderm, and endoderm [2]. It most commonly arises from the gonads followed by extragonadal sites: mediastinal, retroperitoneal, presacral, and coccygeal areas [3]. Retroperitoneal teratoma accounts for only 4% of all teratomas and is commonly found in children [2]. In adults, retroperitoneal teratoma accounts for 1–11% of retroperitoneal tumors [4]. The majority of the patients were asymptomatic. Because malignancy is observed in about 25% of the cases [4], surgical resection should be performed when suspecting retroperitoneal teratoma [5].

It is challenging to clinically differentiate retroperitoneal teratoma from adrenal myelolipoma when a mass which is composed of fat and calcification is located closed to the adrenal gland, as was seen in the present case. Yumura et al. proposed several characteristics to differentiate teratoma from myelolipoma in terms of radiological findings on calcification, fat, and solid components [6]. According to literature review by these authors, teratoma tends to show rough and bulky calcifications whereas myelolipoma has punctate calcifications. Teratoma generally shows contrast-enhanced solid components but myelolipoma does not. In addition, myelolipoma is often occupied by fatty components by greater than 80% whereas teratoma is lesser by up to 50% [6]. In the present case, the tumor had a relatively rough calcification and fat components at about 50 volume percent, suggesting the diagnosis of teratoma. However, no solid component was observed on either CT or MRI, and thus it caused difficulty to make preoperatively diagnosis of retroperitoneal teratoma.

Generally, adrenal myelolipoma does not need surgical excision because of its benign nature. However, surgical removal is needed when the differential diagnosis from retroperitoneal teratoma is difficult. In the present case, curative resection of retroperitoneal teratoma was eventually made via a minimally invasive retroperitoneoscopic approach using the RoboSurgeon system [1]. Minimally invasive surgery yields earlier recovery and less morbidity than conventional open surgery when performing adrenalectomy [7]. Thus, minimally invasive surgery should be considered as a treatment option for a case of an adrenal mass difficult to radiologically distinguish retroperitoneal teratoma from adrenal myelolipoma.

## 4. Conclusions

Retroperitoneal teratoma is a relatively rare but clinical important tumor which needs curative resection due to a potential risk of malignancy. Considering the diagnostic difficulty of retroperitoneal teratoma by radiological imaging, surgical resection via a minimally invasive approach may be a viable treatment option in a case of an adrenal mass difficult to distinguish retroperitoneal teratoma from adrenal myelolipoma.

## Abbreviations

CT: Computed tomography
MRI: Magnetic resonance imaging.

## References

[1] Kihara K., Fujii Y., Masuda H. (2012). New three-dimensional head-mounted display system, TMDU-S-3D system, for minimally invasive surgery application: procedures for gasless single-port radical nephrectomy. *International Journal of Urology*.

[2] Azizkhan R. G., Caty M. G. (1996). Teratomas in childhood. *Current Opinion in Pediatrics*.

[3] Polo J. L., Villarejo P. J., Molina M. (2004). Giant mature cystic teratoma of the adrenal region. *American Journal of Roentgenology*.

[4] Gatcombe H. G., Assikis V., Kooby D., Johnstone P. A. S. (2004). Primary retroperitoneal teratomas: a review of the literature. *Journal of Surgical Oncology*.

[5] Leandros E., Alexakis N., Konstadoulakis M., Albanopoulos K., Dikoglou C., Bramis J. (2005). Postchemotherapy resection of a primary mature malignant retroperitoneal teratoma in an adult: report of a case. *Surgery Today*.

[6] Yumura Y., Chiba K., Urushibara M., Saito K., Hirokawa M. (2000). A case of retroperitoneal teratoma difficult to distinguish from adrenal myelolipoma. *Hinyokika Kiyo*.

[7] Hazzan D., Shiloni E., Golijanin D., Jurim O., Gross D., Reissman P. (2001). Laparoscopic vs open adrenalectomy for benign adrenal neoplasm. *Surgical Endoscopy*.

